# Clinical Effectiveness and Safety Comparison between Reduced Rivaroxaban Dose and Dual Antiplatelet Therapy for Nonvalvular Atrial Fibrillation Patients Following Percutaneous Left Atrial Appendage Closure: A Prospective Observational Study

**DOI:** 10.31083/j.rcm2411335

**Published:** 2023-11-27

**Authors:** Xiaoye Li, Qinchun Jin, Yao Yao, Xiaochun Zhang, Qianzhou Lv

**Affiliations:** ^1^Department of Pharmacy, Zhongshan Hospital, Fudan University, 200032 Shanghai, China; ^2^Department of Cardiology, Zhongshan Hospital, Fudan University, 200032 Shanghai, China

**Keywords:** clinical effectiveness and safety, reduced rivaroxaban dose, dual antiplatelet therapy, nonvalvular atrial fibrillation, left atrial appendage closure

## Abstract

**Background:**

Device-related thrombosis (DRT) after successful closure implantation on left atrial appendage (LAA) was considered as a major challenge and optimal strategy on antithrombotic therapy remains to be solved. This study was performed to compare the clinical effectiveness and safety of reduced rivaroxaban dose (RRD) and dual antiplatelet therapy (DAPT) after left atrial appendage closure (LAAC) implantation with the Watchman device.

**Methods:**

After successful LAAC, consecutive participants were medicated with a standard DAPT or RRD. The primary endpoints included DRT, thrombosis events (TE), and bleeding events that were documented during a 12-month follow-up period.

**Results:**

767 patients (DAPT: n = 140; RRD: n = 627) were initially included. After propensity score matching (PSM), 140 patients treated with DAPT and 280 patients with RRD were included in each group with similar baseline information, thromboembolic and bleeding risk factors, cardiovascular risk factors and concomitant medication. In the RRD group, 193 patients were on rivaroxaban 15 mg ($R^{15}$) and 47 received rivaroxaban 10 mg ($R^{10}$). The incidence of DRT was documented in 12 (9.3%) patients in the DAPT group and 3 (6.3%) in $R^{10}$ and 7 (3.0%) in $R^{15}$ (log-rank *p* = 0.050). DAPT subgroups were more likely to experience shorter time to DRT as compared to $R^{15}$ ($R^{15}$ *vs*. DAPT hazard ratio (HR) = 0.334, *p* = 0.015, 95% CI: 0.131–0.850). The median length of DRT in the $R^{15}$ group was significantly lower than that of the DAPT group (1.721 [1.610–1.818] mm *vs*. 1.820 [1.725–1.925] mm, *p* = 0.029). Compared with the unadjusted estimated rates of ischemic events for patients with similar congestive heart failure, hypertension, age ≥75 years, diabetes mellitus, prior stroke or transient ischemic attack or thromboembolism, vascular disease, age 65–74 years, sex category (${\mathrm{CHA}}_{2}$${\mathrm{DS}}_{2}$-${\mathrm{VAS}}_{\text{c}}$) scores, a significant decrease of 68.6% in ischemic stroke rates was noted in the $R^{15}$ group, which contributed to a 54.9% reduction of overall thromboembolic events. The overall minor bleeding was not significantly different amongst the three groups (*p* = 0.944). Procedural bleeding was more common in the DAPT group, as compared with the $R^{10}$ and $R^{15}$ groups.

**Conclusions:**

After successful closure implantation, long-term RRD significantly reduced the DRT and TE occurrence compared with DAPT.

## 1. Introduction

Left atrial appendage closure (LAAC) has been currently proven to be effective and safe in stroke prevention among patients with non-valvular atrial fibrillation (NVAF) [1, 2]. Long-term follow-up revealed that LAAC significantly reduced the mortality of cardiovascular disease and all-cause mortality [3]. LAAC was regarded as an effective and safe alternative to oral anticoagulation (OAC) in thromboembolic (TE) prevention related to NVAF among patients contraindicated to long-term anticoagulation [4, 5]. Nowadays, thrombus development on the device after successful device insertion was considered as a major challenge with a reported incidence ranging from 3% to 5% of cases, which was considered as an increased thrombotic risk [6, 7].

Several antithrombotic strategies had been adopted for thrombus prevention after LAAC while endothelialization of the device is achieved. Current guidelines recommended a 45-day period of anticoagulation with a direct oral anticoagulant (DOAC) or warfarin after LAAC followed by dual antiplatelet therapy (DAPT) up to 6 months and then aspirin (100 mg qd) alone for life [8]. Nonetheless, concerns regarding bleeding risks and delayed device-related thrombosis (DRT) have prompted interest in exploring alternative antithrombotic regimens [9, 10].

Reduced rivaroxaban dose (RRD) has gained increasing attention as a potential alternative anticoagulation strategy for patients undergoing LAAC [11]. RRD, which involves a lower dose of rivaroxaban than typically used for anticoagulation, has shown promise as a potential alternative for reduction of DRT and thrombosis events (TE) without increasing bleeding risks [12, 13]. Clinical trials indicated that RRD has been proposed as a potentially effective approach to reduce the incidence of DRT without compromising safety [14, 15]. In the sub-analysis of J-ROCKET AF, the thrombotic and bleeding occurrence of RRD (10 mg) was consistent in patients with preserved renal function and moderate renal impairment, which confirmed the validity of RRD (10 mg) once daily for east Asia population with moderate renal impairment [16]. However, the efficacy and safety of RRD as a post-LAAC anticoagulation strategy have not been well studied.

Therefore, the main purpose of this study was to investigate the effectiveness and safety of RRD as a post-LAAC anticoagulation strategy for DRT and TE prevention without an increased bleeding risk during 1-year follow-up. The findings of this study may provide insights into the potential benefits and limitations of RRD as an antithrombotic regimen for LAAC.

## 2. Methods

### 2.1 Study Population and Design

This was a prospective, observational and single center study including consecutive eligible participants following percutaneous LAAC between September 2016 and September 2020. Ethics approval of antithrombotic protocols was granted by the Ethics Committee of Zhongshan Hospital, Fudan University. Patients eligible for Watchman (Boston Scientific, Natick, MA, USA) implantation met the following inclusion criteria: (1) age >18; (2) diagnosis as NVAF; (3) the potential ischemic stroke score (${\mathrm{CHADS}}_{2}$) ≥2 or a congestive heart failure, hypertension, age ≥75 years, diabetes mellitus, prior stroke or transient ischemic attack or thromboembolism, vascular disease, age 65–74 years, sex category (${\mathrm{CHA}}_{2}$${\mathrm{DS}}_{2}$-${\mathrm{VAS}}_{\text{c}}$) score ≥3; (4) intolerant of long-term anticoagulants or at higher risk for bleeding. Participants who met the following criteria were excluded: (1) receiving long-term DAPT prior to Watchman implantation; (2) participants who were transferred to surgery due to the complications of LAAC procedures; (3) AF ablation planned during the follow-up.

There is currently concern regarding post-LAAC antithrombotic regimens. However, there is limited evidence on DRT and bleeding prevention. Therefore, this observational study sought to provide further data regarding the antithrombotic strategy among Chinese patients following LAAC. The study was not randomized; instead, the antithrombotic protocol was determined by the implanting physicians’ judgment. Subsequently, the patients were divided into three groups based on their prescribed antithrombotic plans, which were at the discretion of the physician. The RRD group comprised participants in the rivaroxaban 10 mg ($R^{10}$) or 15 mg ($R^{15}$), who were initially medicated with 45 days of rivaroxaban 10 mg or 15 mg after operation. Subsequently these participants were switched to DAPT (aspirin 100 mg plus clopidogrel 75 mg) after confirming the adequate closure stability and no significant peridevice leak at 45-day trans-esophageal echocardiography (TEE) examination. After 6-month following TEE confirmation, mono-antiplatelet was continued indefinitely. Another group comprised patients with DAPT, who were prescribed aspirin (100 mg) and clopidogrel (75 mg) for 180 days after closure implantation and then long-term aspirin therapy. If the 45-day TEE revealed DRT, the antithrombotic strategy was switched to full dose rivaroxaban (20 mg qd) until the second TEE confirmation a total elimination of DRT.

### 2.2 Device Implantation Procedure

The LAAC device implantation had been described in detail [17]. The procedure was performed under general anesthesia with fluoroscopy and intracardiac echocardiography guidance. The post-implant anti-thrombotic regimen was individualized, and left to physician discretion. Participants were discharged after observation with no periprocedural complication. A routine TEE examination was performed 45 days after device implantation to determine the presence of significant residual flow (>5 mm) or DRT.

### 2.3 In- and Out-of-Hospital Follow-Up

Detailed demographic and baseline clinical parameters were recorded from hospital information systems (HIS). ${\mathrm{CHA}}_{2}$${\mathrm{DS}}_{2}$-${\mathrm{VAS}}_{\text{c}}$ and hypertension, abnormal renal or liver function, stroke, bleeding, labile international normalized ratio, elderly, drugs or alcohol (HAS-BLED) score were determined in each patient for risk stratification of potential thromboembolism and bleeding risks. Laboratory parameters including liver, renal function and coagulation were also recorded. TEE was conducted to rule out a cardiac effusion post procedure.

Routine outpatient follow-ups performed for each enrolled participant included up to 3 repeated TEE examinations scheduled approximately 45 days, 180 days and yearly post procedure for the presence of DRT. Out-patient visits and trans-telephonic clinical evaluations were conducted every 3 months during the 1-year follow-up. All the follow-up TEE images and recordings were reviewed by one physician and participants who did not complete the follow-up examination were excluded from the final analysis.

### 2.4 Clinical Endpoints

The primary clinical endpoint was a composite of effective and safety characteristics of each strategy. The efficacy endpoints were as followings: (1) DRT defined as a well-circumscribed and uniformly echo-dense mass lying on the closure, measured by TEEs, (2) TE events including stroke or transient ischemic attack (TIA) determined on magnetic resonance imaging (MRI) or computed tomography (CT), peripheral thromboembolism, pulmonary embolism and venous thromboembolism. The safety endpoints included clinical major and non-major bleeding complications defined according to the guidance of the International Society on Thrombosis and Haemostasis [18]. The definition of major bleeding in the study involved a decrease in the hemoglobin level of no less than 20 g/L, transfusion of two or more units of blood, or symptomatic bleeding that affected a critical organ. Clinically significant non-major bleeding was defined as bleeding that necessitated medical attention from a healthcare professional, a higher level of intensive care, or an on-site evaluation.

### 2.5 Sample Size

The sample size was calculated based on the lower hospitalization rate of confirmed DRT and TE with long-term RRD compared with a standard antiplatelet therapy. Using PASS statistical software (version 11.0; NCSS, LLC. Kaysville, UT, USA), a class I error rate (α) of 0.05 and a statistical power of 90% (class II error rate β = 0.1) were selected. To account for a 10% attrition rate, the study sought to enroll a minimum of 240 eligible participants.

### 2.6 Statistical Analyses

Continuous variables were presented as mean ± standard deviation (SD) and compared by the Mann-Whitney *U* tests or Student *t*-tests between the two groups mainly dependent on the normal distribution. Categorical variables were presented as frequencies or percentages n (%) and analyzed using χ^2^ or Fisher’s precision probability tests.

The baseline characteristics comparison between groups was conducted using appropriate statistical tests, such as *t*-tests and χ^2^ tests/Fisher’s precision probability test.

The primary efficacy and safety variables were the cumulative occurrence of confirmed DRT, TE, and bleeding complications, for each enrolled patient during the follow-up period. Kaplan-Meier curves were performed to illustrate the time-to-first thrombosis or bleeding, and log-rank tests were used to compare these curves. Statistical analysis was performed using SPSS software (version 22.0; IBM Corp., Armonk, NY, USA), and a *p* value of 0.05 was considered statistically significant.

## 3 Results

### 3.1 Baseline Characteristics of Study Populations

**Fig. 1.**
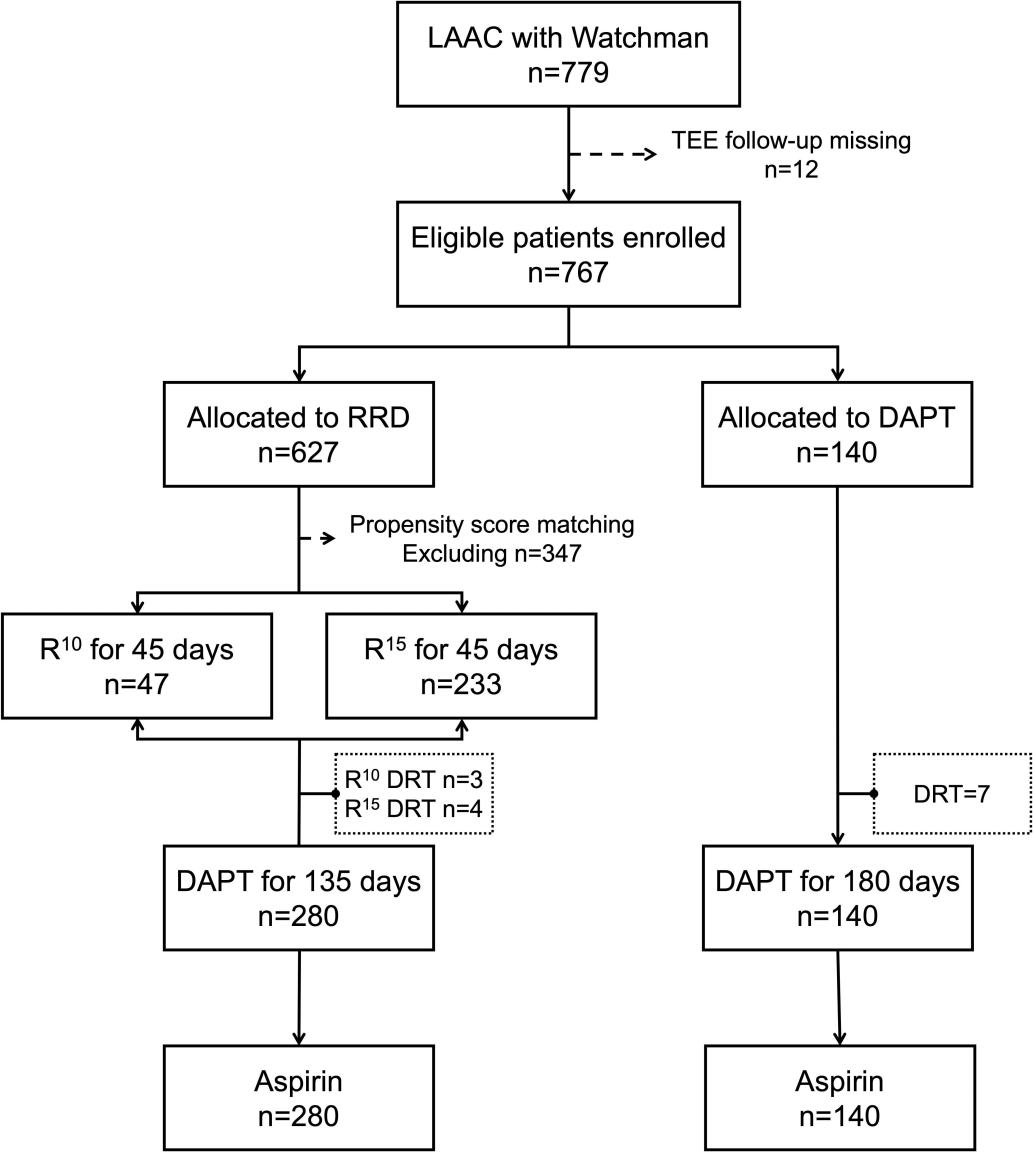
**Enrollment flow chart of patients**. LAAC, left atrial appendage closure; TEE, trans-esophageal echocardiography; RRD, reduced rivaroxaban doses; DAPT, dual antiplatelet therapy; $R^{10}$, rivaroxaban 10 mg; $R^{15}$, rivaroxaban 15 mg; DRT, device-related thrombosis.

This cohort study initially enrolled 779 patients following successful Watchman implantation between December 2017 and December 2021. A total of 12 participants (DAPT group: n = 6 [0.8%]; RRD group: n = 6 [0.8%]) were finally excluded from the study because their follow-up TEEs were completed at a different clinical institution and no images were provided for review. Ultimately, 767 patients (DAPT group: n = 140; RRD group: n = 627; Mean age 68.0 ± 9.0, male 478 (62.3%), Median ${\mathrm{CHA}}_{2}$${\mathrm{DS}}_{2}$-${\mathrm{VAS}}_{\text{c}}$ score 4; Median HAS-BLED score 2) were included in the analysis and followed for 12 months. Among participants in the RRD group, the anticoagulant regimen was rivaroxaban 15 mg once a day in 551 (87.9%) patients and 10 mg once a day in 76 (12.1%). The progression of anticoagulation therapy for post-LAAC operation is summarized in Fig. 1.

Baseline information, cardiovascular risk factors, potential thromboembolic and bleeding risks, and concomitant medication are presented in Table 1. There was a higher percentage of higher HAS-BLED score in participants taking DAPT. After propensity score matching (PSM) with 1:2 ratio (140 patients for DAPT and 280 patients for RRD), the two subgroups were not significantly different in baseline information, cardiovascular risk factors, laboratory indicators and predetermined stroke and bleeding risk.

**Table 1.** **Baseline demographic and clinical characteristics between RRD and DAPT groups**.

Variables		Before matching				After matching			
		All (N = 767)	RRD (n = 627)	DAPT (n = 140)	*p* value	All (N = 420)	RRD (n = 280)	DAPT (n = 140)	*p* value
Age, y		68.0 ± 9.0	67.9 ± 9.2	68.3 ± 8.3	0.639	68.8 ± 8.3	69.0 ± 8.3	68.3 ± 8.3	0.418
Male		478 (62.3)	391 (62.4)	87 (62.1)	0.962	267 (63.6)	180 (64.3)	87 (62.1)	0.667
${\mathrm{CHA}}_{2}$${\mathrm{DS}}_{2}$-${\mathrm{VAS}}_{\text{c}}$ score		3.5 ± 1.8	3.5 ± 1.8	3.7 ± 2.0	0.355	3.7 ± 1.9	3.7 ± 1.9	3.7 ± 2.0	0.844
	≤3	372 (48.5)	309 (49.3)	63 (45.0)	0.359	190 (45.2)	127 (45.4)	63 (45.0)	0.945
	4	169 (22.0)	141 (22.5)	28 (20.0)	0.521	86 (20.5)	58 (20.7)	28 (20.0)	0.864
	≥5	226 (29.5)	177 (28.2)	49 (35.0)	0.112	144 (34.3)	95 (33.9)	49 (35.0)	0.827
HAS-BLED score		2.5 ± 1.2	2.4 ± 1.2	3.2 ± 1.2	0.001	3.1 ± 1.2	3.0 ± 1.2	3.2 ± 1.2	0.092
	≤2	391 (51.0)	352 (56.1)	39 (27.9)	0.001	115 (27.4)	76 (27.1)	39 (27.9)	0.877
	3	217 (28.3)	170 (27.1)	47 (33.6)	0.125	154 (36.7)	107 (38.2)	47 (33.6)	0.352
	4	121 (15.8)	81 (12.9)	40 (28.6)	0.001	113 (26.9)	73 (26.1)	40 (28.6)	0.586
	≥5	38 (5.0)	224 (3.8)	14 (10.0)	0.002	38 (9.0)	24 (8.6)	14 (10.0)	0.630
Risk factors for stroke and bleeding									
CHF		10 (1.3)	8 (1.3)	2 (1.4)	1	5 (1.2)	3 (1.1)	2 (1.4)	1.000
Hypertension		488 (63.6)	398 (63.5)	90 (64.3)	0.857	293 (69.8)	203 (72.5)	90 (64.3)	0.084
≥75 years of age		179 (23.3)	144 (23.0)	35 (25.0)	0.607	104 (24.8)	69 (24.6)	35 (25.0)	0.936
65–74 years of age		345 (45.0)	283 (45.1)	62 (44.3)	0.855	202 (48.1)	140 (50.0)	62 (44.3)	0.269
Diabetes mellitus		160 (20.9)	124 (19.8)	36 (25.7)	0.118	79 (18.8)	50 (17.9)	29 (20.7)	0.480
History of stroke/TIA		326 (42.5)	266 (42.4)	60 (42.9)	0.925	186 (44.3)	126 (45.0)	60 (42.9)	0.677
	Stroke	282 (36.8)	223 (35.6)	59 (42.1)	0.145	179 (42.6)	120 (42.9)	59 (42.1)	0.889
	TIA	49 (6.4)	43 (6.9)	6 (4.3)	0.26	17 (4.0)	8 (2.9)	9 (6.4)	0.080
Vascular disease		407 (53.1)	329 (52.5)	78 (55.7)	0.487	228 (54.3)	150 (53.6)	78 (55.7)	0.678
	Renal Dysfunction	44 (5.7)	32 (5.1)	12 (8.6)	0.111	37 (8.8)	25 (8.9)	12 (8.6)	0.903
	Liver Dysfunction	71 (9.3)	55 (8.8)	16 (11.4)	0.327	53 (12.6)	37 (13.2)	16 (11.4)	0.603
History of major bleeding		56 (7.3)	41 (6.5)	15 (10.7)	0.086	50 (11.9)	35 (12.5)	15 (10.7)	0.594
	Intracranial bleeding	33 (4.3)	25 (4.0)	8 (5.7)	0.363	30 (7.1)	22 (7.9)	8 (5.7)	0.421
	GI bleeding	13 (1.7)	10 (1.6)	3 (2.1)	0.715	10 (2.4)	7 (2.5)	3 (2.1)	1.000
	Other	11 (1.4)	7 (1.1)	4 (2.9)	0.123	11 (2.6)	7 (2.5)	4 (2.9)	1.000
History of minor bleeding		21 (2.7)	14 (2.2)	7 (5.0)	0.07	17 (4.0)	10 (3.6)	7 (5.0)	0.484
	GI bleeding	4 (0.5)	3 (0.5)	1 (0.7)	0.554	3 (0.7)	2 (0.7)	1 (0.7)	1.000
	Epistaxis	3 (0.4)	2 (0.3)	1 (0.7)	0.454	2 (0.5)	1 (0.4)	1 (0.7)	1.000
	Other	13 (1.7)	9 (1.4)	4 (2.9)	0.271	6 (2.1)	2 (1.4)	4 (2.9)	0.684
Labile INR		36 (4.7)	29 (4.6)	7 (5.0)	0.85	32 (7.6)	25 (8.9)	7 (5.0)	0.153
Alcohol		43 (5.6)	38 (6.1)	5 (3.6)	0.247	33 (7.9)	27 (9.6)	6 (4.3)	0.054
CAD		122 (15.9)	93 (14.9)	29 (20.7)	0.085	68 (16.2)	39 (13.9)	29 (20.7)	0.075
LVEF, %		63.1 ± 6.8	63.1 ± 6.8	62.7 ± 6.8	0.471	63.0 ± 6.9	63.1 ± 7.1	62.7 ± 6.7	0.575

Values are mean ± SD, n (%). RRD, rivaroxaban dose; DAPT, dual antiplatelet therapy; CAD, coronary artery disease; ${\mathrm{CHA}}_{2}$${\mathrm{DS}}_{2}$-${\mathrm{VAS}}_{\text{c}}$, congestive heart failure, hypertension, age ≥75 years, diabetes mellitus, prior stroke or transient ischemic attack or thromboembolism, vascular disease, age 65–74 years, sex category; CHF, congestive heart failure; INR, international normalized ratio; LVEF, left ventricular ejection fraction; GI, gastrointestinal; HAS-BLED, hypertension, abnormal renal or liver function, stroke, bleeding, labile international normalized ratio, elderly, drugs or alcohol; TIA, transient ischemic attack.

### 3.2 Efficacy Endpoints Evaluation

The RRD group had a 12-month median (interquartile range (IQR): 11–14) follow-up and the DAPT group had a 13-month (IQR: 11–15) median follow-up. In the RRD group, 193 were on $R^{15}$ and 47 on $R^{10}$. In the first TEEs (within 2 days after LAAC), peri-device leaks >5 mm were recorded in 4 cases ($R^{15}$ (n = 1), $R^{10}$ (n = 1) and DAPT (n = 2)). A second TEE was performed to confirm the presence of leaks among these patients, who were subsequently scheduled for percutaneous LAA leak closure. The primary efficacy endpoints were defined as a function of both the presence of DRT as well as the occurrence of thrombosis events. Early-phase formation of DRT was investigated, as reflected by the incidence of DRT and thrombus size. These patients were prescribed full-dose rivaroxaban (20 mg qd).

**Fig. 2.**
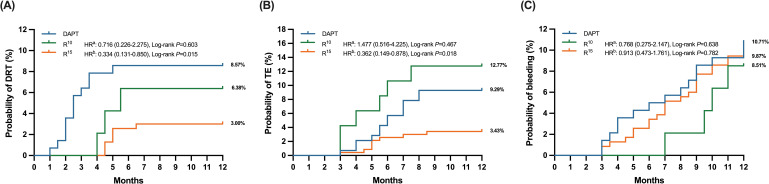
**Time to clinical events in antithrombotic treated patients, stratified into three subgroups (DAPT, $R^{10}$ and $R^{15}$) according to the different antithrombotic strategy**. (A) referred as the comparison between DAPT and $R^{10}$. (B) referred as the comparison between (C) DAPT and $R^{15}$. (A) Kaplan-Meier survival curve of device-related thrombus (DRT), (B) Kaplan-Meier survival curve of thromboembolic (TE) events, (C) Kaplan-Meier survival curve of bleeding events. DAPT, dual antiplatelet therapy; $R^{10}$, rivaroxaban 10 mg; $R^{15}$, rivaroxaban 15 mg; HR, hazard ratio.

The incidence of DRT was documented in 12 (9.3%) patients in DAPT, 3 (6.3%) in $R^{10}$ and 7 (3.0%) in $R^{15}$ (log-rank *p* = 0.050), which indicated a higher DRT incidence in DAPT than that of $R^{10}$ and $R^{15}$. A clear causal relationship between the TE and DRT could be established in 3 cases which were identified as ischemic strokes. As a result of switching to rivaroxaban therapy at full dose (20 mg), the DRTs were successfully managed. In the whole cohort of antithrombotic treated patients, DAPT subgroups were more likely to experience shorter time to DRT ($R^{10}$ *vs*. DAPT, HR = 0.716, *p* = 0.603, 95% CI: 0.226–2.275; $R^{15}$ *vs*. DAPT HR = 0.334, *p* = 0.015, 95% CI: 0.131–0.850), as demonstrated in Fig. 2A.

In the DAPT group, a total of 14 patients (10.0%) experienced TEs in terms of ischemic stroke or systemic embolism during the follow-up period compared with 6 patients (12.7%) in the $R^{10}$ and 8 (3.4%) in the $R^{15}$ matched group. Based on Kaplan-Meier survival analysis, TE reduction was significantly more favorable in the $R^{15}$ group ($R^{15}$ *vs*. DAPT HR = 0.362, *p* = 0.018, 95% CI: 0.149–0.878, Fig. 2B).

**Fig. 3.**
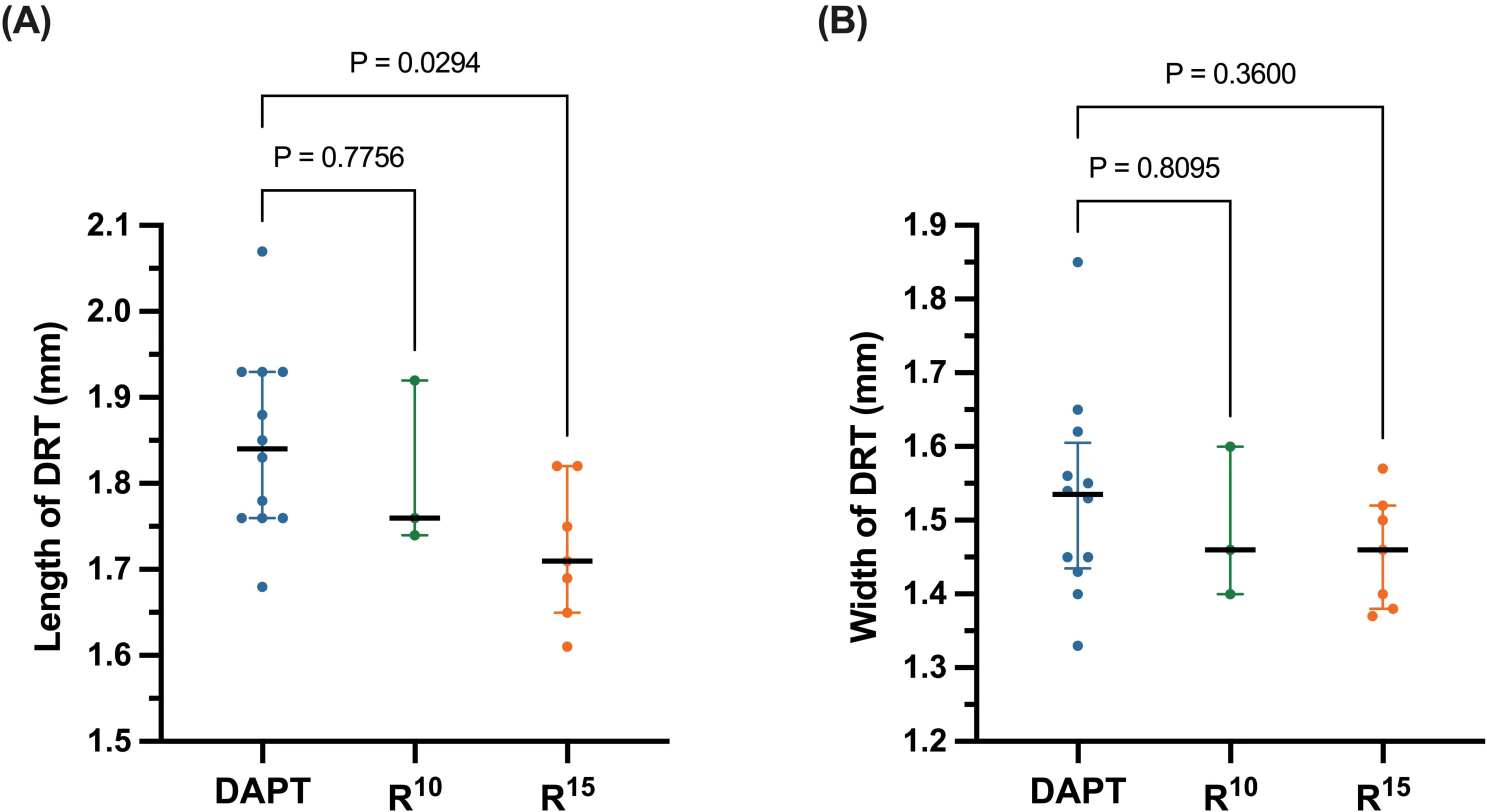
**(A) Length and (B) width of device-related thrombus (DRT) evaluated with transesophageal echocardiography**. The solid black lines medians of each subgroup, while the error bars represent the interquartile range. DAPT, dual antiplatelet therapy; $R^{10}$, rivaroxaban 10 mg; $R^{15}$, rivaroxaban 15 mg.

The median length of DRT in the $R^{15}$ group was significantly lower than that of the DAPT group (1.721 [1.610–1.818] mm *vs*. 1.820 [1.725–1.925] mm, *p* = 0.029), while no significant difference was detected between $R^{10}$ and DAPT (1.806 [1.740–1.924] mm *vs*. 1.820 [1.725–1.925] mm, *p* = 0.775), as shown in Fig. 3A. No significant difference in DRT width was observed among the three groups ($R^{10}$ 1.486 (1.402–1.620) *vs*. DAPT 1.520 [1.400–1.610], *p* = 0.809; $R^{15}$ 1.457 (1.372–1.578) *vs*. DAPT 1.520 [1.400–1.610], *p* = 0.360, Fig. 3B).

**Fig. 4.**
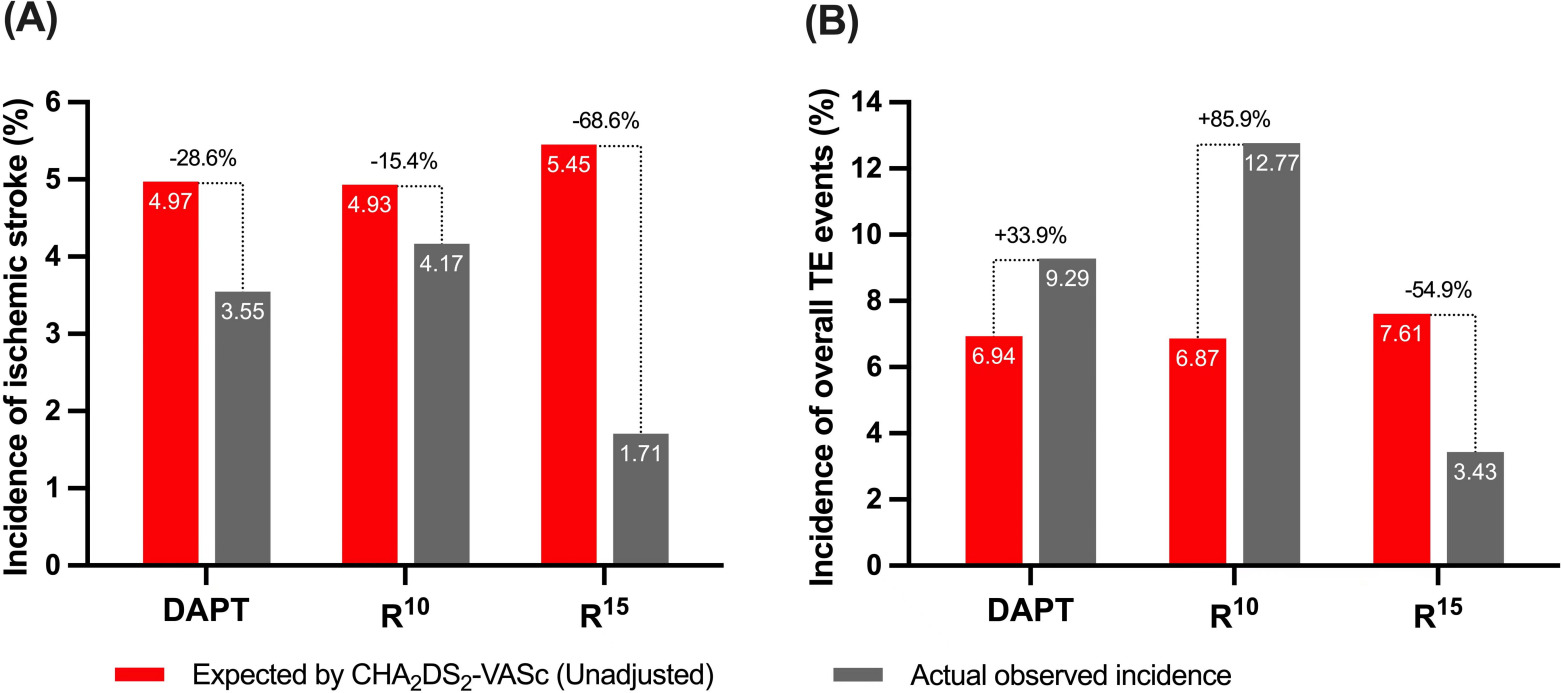
**(A) Annualized ischemic stroke and (B) TE event rates after implantation vs expected (unadjusted) rates estimated based on the ${\mathrm{CHA}}_{2}$${\mathrm{DS}}_{2}$-${\mathrm{VAS}}_{\text{𝐜}}$ (congestive heart failure, hypertension, age >75 years, diabetes mellitus, prior stroke or transient ischemic attack or thromboembolism, vascular disease, age 65–74 years, sex category) score of the 3 study groups**. DAPT, dual antiplatelet therapy; $R^{10}$, rivaroxaban 10 mg; $R^{15}$, rivaroxaban 15 mg; TE, thromboembolic.

Compared with the unadjusted estimated rates of ischemic events for patients with similar ${\mathrm{CHA}}_{2}$${\mathrm{DS}}_{2}$-${\mathrm{VAS}}_{\text{c}}$ scores, a significant decrease of 68.6% in ischemic stroke rates was noted in the $R^{15}$ group, while slight insignificant reductions of 28.6% and 15.4% was observed in the DAPT and $R^{10}$ groups (Fig. 4A). In the whole cohort study, $R^{15}$ contributed a 54.9% reduction of overall TE events, as shown in Fig. 4B.

### 3.3 Safety Endpoints Evaluation

Details of the bleeding events in the entire patient cohort are reported in Table 2. No major bleeding was documented throughout the 12-month follow-up period. The rate of overall minor bleeding was not significantly different amongst the groups (8.5% among $R^{10}$ patients, 9.9% among $R^{15}$ patients and 10.7 among DAPT patients, (*p* = 0.944).

**Table 2.** **Bleeding complications comparison among $R^{10}$, $R^{15}$ and DAPT groups**.

Bleeding complications, n (%)		$R^{10}$	${\mathrm{R1}}^{5}$	DAPT	*p* value
Overall bleeding events		4 (8.5%)	23 (9.9%)	15 (10.7%)	0.944
	GI bleeding	1 (2.1%)	5 (2.1%)	3 (2.1%)	1.000
	Hematuria	1 (2.1%)	4 (1.7%)	1 (0.7%)	0.586
	Operation site hemorrhage	0 (0.0%)	4 (1.7%)	2 (1.4%)	1.000
	Bleeding gums	1 (2.1%)	6 (2.6%)	4 (2.9%)	1.000
	Skin ecchymosis	1 (2.1%)	4 (1.7%)	5 (3.6%)	0.520
PLT <125 × ${\mathrm{10}}^{9}$/L		4 (8.5%)	10 (8.1%)	15 (10.7%)	0.745
Male: Hb <120 g/L		5 (10.6%)	11 (7.3%)	16 (11.4%)	0.748
Female: Hb <110 g/L					
PT >13 s		12 (25.5%)	62 (26.6%)	32 (22.8%)	0.856

GI, Gastrointestinal; PLT, platelet; Hb, hemoglobin; PT, prothrombin time; DAPT, dual antiplatelet therapy; $R^{10}$, rivaroxaban 10 mg; $R^{15}$, rivaroxaban 15 mg. *p*-value represented with interaction.

Table 2 shows the accumulated anticoagulation-related complications and coagulation function tests among the groups. There was no significant reduction in levels of platelet (PLT), hemoglobin (Hb), or prothrombin time (PT) among $R^{10}$, $R^{15}$ and DAPT groups (*p* > 0.05). Based on Kaplan-Meier survival curve analysis, procedural bleeding was more common in the DAPT group, as compared with the $R^{10}$ and $R^{15}$ groups (Fig. 2), however, there was no significant difference among three groups.

## 4. Discussion

In this study, we prospectively investigated the clinical efficacy and safety between RRD and DAPT after successful LAAC implantation. The following are the main findings of the study. First, anticoagulation long term RRD led to a significant reduction of DRT and TE compared to DAPT. Second, long-term rivaroxaban provided more effective and safer thrombus prevention, when compared with unadjusted, estimated rates of ischemic stroke and TE for patients with similar ${\mathrm{CHA}}_{2}$${\mathrm{DS}}_{2}$-${\mathrm{VAS}}_{\text{c}}$.

Similar to all medical devices implanted into the body, a longer implantation time beyond 90 days is expected to enable complete endothelialization for occluders post LAAC when exposed to circulating blood [19, 20]. Thrombosis formation might occur on the exposed device and adequate antithrombotic regimens are required for DRT prevention. Currently, pharmacological regimens following successful LAAC implantation were mainly dependent on strategies from recent clinical studies [21, 22]. Previous studies indicated short-term DAPT adoption followed by long-term aspirin could prevent DRT and TE events [23, 24]. Although antiplatelet therapy has confirmed the efficacy for thrombosis prevention after stent implantation, substantial variation still remains for the selection of the appropriate antithrombotic strategy for LAAC implantation. In this study we sought to compare the clinical efficacy and safety between RRD and standard antiplatelet strategy following LAAC. Some novel observations could be made based on the data derived from this study.

It is important to identify anticoagulants that can prevent the occurrence of DRT and TE. Currently, there is limited data regarding the correlation between antithrombotic strategies and thrombus on closure devices. In this study, the scheduled out-patient visits and trans-telephonic clinical evaluations were frequently conducted 6 months after discharge to help reduce the occurrence of thromboembolic events. Our results documented a lower DRT rate in RRD as compared to DAPT, which was similar to those in previously published studies from other groups [14, 25]. In one propensity matched comparison with Watchman closure implantation patients, the 6-month cumulative DRT occurrence was lower in DAPT as compared to half-Dose DOAC (3.4% *vs*. 0.0%), which was similar with our findings [14]. Another multicenter study with patients undergoing LAAC implantation indicated that DOACs proved to be a feasible and safe alternative antithrombotic regimen to warfarin for DRT and thromboembolic prevention after LAAC implantation, without increasing the risk of bleeding [14].

Activation of the coagulation system and enhanced thrombin generation without platelet aggregation were associated with DRT formation within days after LAAC [26]. In our results, persistent elevation of thrombosis size was also detected in the DAPT group after the procedure, which was consistent with the result of previous prospective studies. A randomized pilot study documented that circulating prothrombin fragments and thrombin-antithrombin complex were numerically lower after rivaroxaban treatment than that with DAPT, which might explain the lower rate of thrombosis and TE following medication with rivaroxaban after successful LAAC [27].

Another important factor for consideration is the safety for long-time anticoagulation among different antithrombotic regimens in patients undergoing LAAC. Our results indicated decreased bleeding occurrence after the initial follow-up, which might be related to more scheduled out-patient visits and clinical evaluations 6 months after discharge. Of note, a reduced DOAC dose was associated with lower bleeding for NVAF patients compared with vitamin K antagonists (VKA) in the east Asia population [28]. One large cohort of east Asia patients showed lower post-extraction bleeding rates with DOAC compared with warfarin (HR: 0.84; 95% CI: 0.54–1.31) [29]. In our study, there was no significant difference in coagulation function tests between the RRD and DAPT groups. Furthermore, our findings suggest that RRD may be a safe alternative to DAPT for short-term antithrombotic therapy after LAAC, without a statistically significant difference observed in bleeding events. However, the optimal antithrombotic regimen for long-term anticoagulation after LAAC remains uncertain, and individualized treatment plans should be developed based on patient-specific factors, such as bleeding risk, thromboembolic risk, comorbidities, and medication interactions. Although the overall minor bleeding rate was 10.0% in our study, the risk for postoperative bleeding was increased in these patients who underwent a percutaneous strategy and were exposed to anticoagulation therapy. Given the concern for bleeding and the need for TE prophylaxis, a minimally invasive surgical strategy such as epicardial LAA occlusion with no further anticoagulation is more favorable to patients at higher risk for bleeding and thrombosis. The rationale for this practice is inferred from the Left Atrial Appendage Occlusion Study (LAAOS III) trial, which demonstrated that LAA resection for AF patients during chest cardiac surgery contributed to a reduction of thrombosis and bleeding risks [30, 31]. As for NVAF patients with end-stage renal failure contraindicated to DOAC merging with high bleeding risk, epicardial LAA occlusion for such specific population might offer a clinical benefit [32]. Multi-disciplinary teams involving anesthesiologists, cardiologists and cardiac surgeons will need to determine which patients can reliably and safely undergo epicardial LAA closure in those patients with a contraindication for anticoagulation or those with anatomical abnormalities which are not conducive to percutaneous LAAO.

### Limitations

The present study has many limitations. Firstly, our study is non-randomized and conducted at a single center, which might restrict the generalizability of the results to other cardiovascular centers and healthcare systems. Secondly, the sample size was relatively small and the follow-up duration of only 12 months may not be sufficient to determine the long-term efficacy and safety of different anticoagulation regimens, which potentially led to a low incidence of DRT. The exclusion of 12 participants due to incomplete follow-up TEEs and failure to provide images for review may have introduced selection bias. Thirdly, our study was followed up only by the cardiology department. Finally, the study only enrolled participants receiving RRD and DAPT, which may limit the generalizability of the findings to other anticoagulants. In conclusion, these limitations highlight the need for further studies with larger sample sizes, more frequent monitoring, and more diverse patient populations to confirm the conclusions and establish the clinical efficacy and safety of different antithrombotic strategies.

## 5. Conclusions

Based on the evidence presented in this cohort study, antithrombotic therapy with RRD may be a promising option for DAPT for reducing the risk of DRT and composite endpoints in patients following successful Watchman implantation. Further randomized controlled trials conducted at multiple centers are needed to compare the safety and efficacy of different antithrombotic regimens in these patients.

## Data Availability

The datasets used and analyzed during the current study are available from the corresponding author on reasonable request.
